# A New Scale for Evaluating the Risks for In-Hospital Falls of Newborn Infants: A Failure Modes and Effects Analysis Study

**DOI:** 10.1155/2010/547528

**Published:** 2010-09-30

**Authors:** Faruk Abike, Sinan Tiras, İlkkan Dünder, Ayfer Bahtiyar, Ozlem Akturk Uzun, Ozlusen Demircan

**Affiliations:** ^1^Department of Obstetrics and Gynecology, Bayindir Hospital, Ankara, 06200 Sogutozu, Turkey; ^2^Department of Neonatology, Bayindir Hospital, Ankara, 06200 Sogutozu, Turkey; ^3^Department of Quality Management, Bayindir Hospital, Ankara, 06200 Sogutozu, Turkey; ^4^Bayindir Hospital, Ankara, 06200 Sogutozu, Turkey

## Abstract

We aimed to develop a new scale for evaluating risks and preventive measures for in-hospital falls of newborn infants, from admission to discharge of the expectant mother. Our study was prepared in accordance with Failure Modes and Effects Analysis criteria. The risks and preventive measures for in-hospital falls of newborns were determined. Risk Priority Numbers (RPNs) were determined by multiplication of the scores of severity, probability of occurrence, and probability of detection. Analyses showed that risks having the highest RPNs were the mother with epidural anesthesia (RPN: 350 point), holding of the baby at the moment of delivery (RPN: 240), and transportation of baby right after delivery (RPN: 240). A reduction was detected in all RPNs after the application of preventive measures. Our risk model can function as a guide for obstetric clinics that need to form strategies to prevent newborn falls.

## 1. Introduction

Operative and vaginal deliveries are among the most commonly performed procedures in hospitals. However, they have a lot of risks, and one may encounter some unpredictable and undesired complications at delivery. The risks for a newborn, including the risks for falling, start with the onset of labor. Falling incidents are particularly encountered at overcrowded delivery and education hospitals, and trauma incidents resulting from falling occur but unfortunately not put on the record. There are a limited number of publications in the literature regarding traumas resulting from in-hospital falls of newborns but there is no scale evaluating the risks and risk reduction measures [1–3]. In a previous study, 14 trauma cases were reported among 888774 deliveries. Trauma incidents of newborn babies resulting from falling were found to be 1.6 per 10.000. Seven of these incidents occurred when the mother holding the infant in a hospital bed or reclining chair fell asleep. Four of the cases occurred in the delivery room, 2 in the hallway while a nurse was wheeling a bassinette, and 1 from an infant swing. No deaths were reported. One patient sustained a depressed skull fracture and was transported to the regional children's hospital [1].

As a part of health care quality and insurance preoccupations some scoring systems have been developed for adult patients [4–7]. The first studies about falling risk were conducted by Morse and Hendrich [5–7], and after that different scoring systems and comparisons of their efficacy have been reported [4, 8–10]. Unfortunately, there are no scoring systems evaluating falls risk in children [11].

Our hospital has been accredited by the Joint Commission International (JCI) in 2006 and our intention is to improve the standards and care quality of the newborn and the mother. The Department of Gynecology and Obstetrics also provides neonatology service in Bayındır Hospitals since the foundation of the hospital. In line with the quality improvement services, we intend to prevent or minimize the risks of newborn falls, although we have not encountered any in-hospital falls in newborns till today. Failure Modes and Effects Analysis (FMEA) is a proactive technique that is most often used to identify and address problems before they occur [12, 13].

In an attempt to identify the risks beforehand, we aimed to develop a new scale for evaluating the risks and preventive measures for in-hospital falls of newborn infants, from admission to the discharge of the expectant mother and the baby, by using FMEA.

## 2. Material and Methods

**Figure 1:**
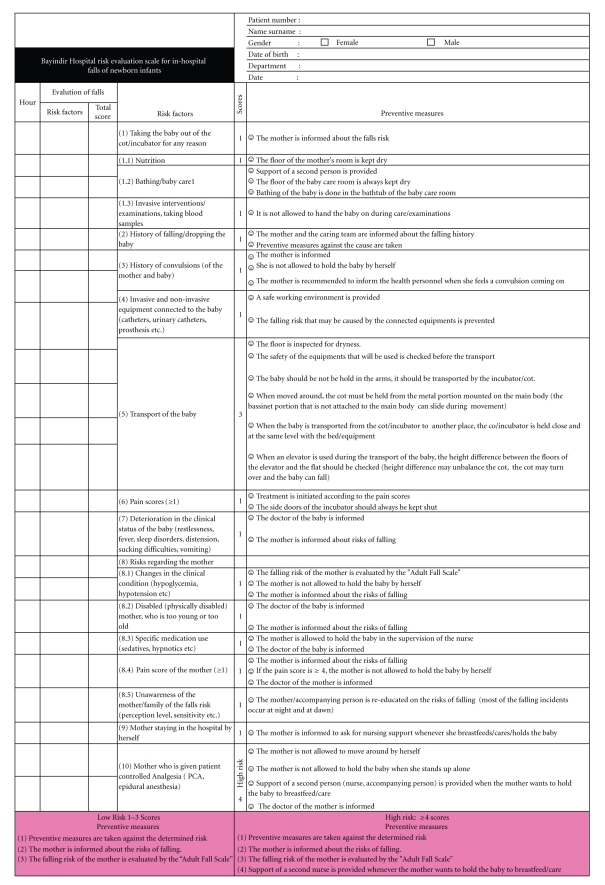
Bayındır Hospital risk evaluation scale for in-hospital falls of newborn infants.

**Figure 2:**
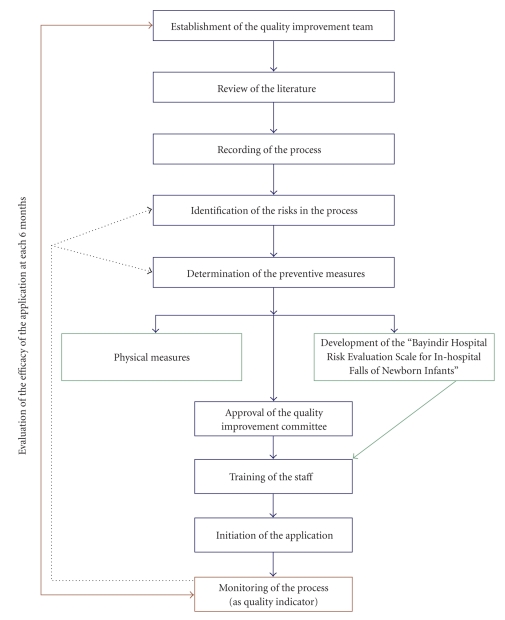
Failure modes and effects analysis workflow algorithm.

Our study was prepared in accordance with the FMEA criteria. A quality improvement team including an obstetrician, a neonatologist, nurses, and quality staff, who were involved in the process, was formed in order to determine the risks and preventive measures for in-hospital falls of newborns. The team worked for 20 hours at 10 sessions, each of which lasted 2 hours, between January and March 2009. Risks, which might be encountered throughout the process, were defined in accordance with FMEA. Firstly, the phases of delivery process, from hospitalization until discharge, were defined as follows: the process before the delivery took place, the delivery process, the transfer of the neonate, and the process of care. The preventive measures, their applicability and efficacy were reviewed. For the probable risks, scores of severity, probability, and predictability were calculated in accordance with the criteria of FMEA. Risk Priority Number (RPN) for each risk was determined by the multiplication of the calculated scores of severity of effect (S), probability of failure (PF), and probability of detection of an existing defect (P). (S × PF × P = RPN). “Bayındır Hospital Risk Evaluation Scale for In-hospital Falls of Newborn Infants” was developed (Figure 1). RPNs were determined twice: before and after the preventive measures. Additionally, the units and the staff that would be involved in the preventive measures were determined (obstetrician, nurse, cleaning personnel, etc.). The algorithm of the process is presented in Figure 2.

## 3. Results

The risks determined for in-hospital falls of newborn infants are presented in Table 1. Scores of severity, probability, and predictability for all risks were calculated in accordance with FMEA scoring system [12], and precautions were determined. The preventive measures and the RPNs before and after the precautions are presented in Table 2. 

**Figure 3:**
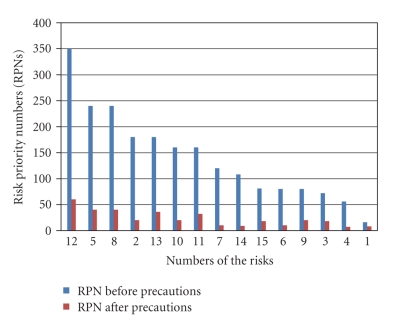
Risk priority numbers before and after the preventive measures. 12: the mother who is given patient-controlled analgesia (PCA) drops the baby when she stands up by herself while no one is near; 5: infant may slip down from the hands of the doctor during delivery; 8: after having the first medical examination (Apgar scores and general condition fine) the newborn may fall due to turning over of the baby cot while it is being transferred to the baby room to be placed in the incubator in the operating room or in the baby cot in the delivery room; 2: birth may occur by standing up and straining of the patient when the cervix is dilated 5 cm or more; 13: during the day the infant is taken to baby room by the baby nurse for checking vital findings, changing diaper, and doctor's examinations; 10: the infant cared for and dressed is placed in the baby cot and handed to the mother; 11: the mother picks up the infant to breastfeed it. Risk factors regarding the mother are analyzed; 7: the newborn may fall while it is taken to the newborn care unit for the first medical examination; 14: blood sample of the infant is taken to test for phenylketonuria and/or bilirubin on the day of discharge. While final preparations are being made the infant may slip down from the baby care unit; 15: after the mother is given the necessary instructions and while they are being discharged, the infant may slip down from the mother's hands; 6: infant may slip down while the umbilical cord is cut; 9: the infant may slip down from the hands of the nurse while it is being transferred to the baby care room in an incubator or baby cot to baby care room; 3: patient may give birth in the preparation room before the doctor arrives; 4: birth may occur while the patient is being transferred to the delivery room; 1: patient is admitted to the service to be prepared for the delivery.

Analyses showed that risks that have the highest RPNs are the mother with patient controlled analgesia (PCA) (RPN: 350 point), holding of the baby at the moment of delivery (RPN: 240), and transportation of the baby right after the delivery (RPN: 240). The other risky conditions were sorted as the patient standing up when the cervix was dilated greater than 5-6 cm (RPN: 180) and the ones that might occur during the basic care of the baby (RPN: 180). After the preventive measures against these risks were implemented rescoring was performed. RPNs of all risks were reduced; the RPN of the mother with PCA dropped to 60, of the baby falling at the moment of delivery and transportation to 40 (Figure 3).

## 4. Discussion

The interest in risk assessment tools and preventive measures for in-hospital falls has been gradually increasing in the recent years by the broad implementation of quality management tools and techniques in the hospitals. Up to date, a number of scales predicting the risks of hospital falls, especially for elderly patients have been developed [6, 7]. However, there is limited number of articles on newborn falling risk and prevention measures [1, 2].

Trauma cases resulting from falling are rare in newborns, but they may be fatal [3]. As most of the cases are not put on the record, information on this issue is limited in the literature. Oregon patient safety commission drew attention on risk of falls to newborns of the mothers with PCA, and they enforced some preventive measures, and the commission indicated that there are a few studies in the literature about in-hospital falls of newborns [2].

Since the accreditation of our hospital, we have been working on quality management in all departments of our hospital. Within the scope of quality of service improvements, we wanted to take precautions against hospital falls, though no cases of newborn falls have been reported in our hospital till now. 

As no scoring system was developed for newborn falls, we used the FMEA method used mainly in other sectors [14, 15] and have been implemented in healthcare in the last decade [16–18]. An FMEA study is not a traditional case control population study. It has some limitations originating primarily from the subjective scoring, while scoring the severity, probability of occurrence and detection, of the risks included in the RPN table. As FMEA, by development of a scale, increases the awareness and sensitivity to known and predictable risks, we think that it is superior to error proofing approach based only on clinical awareness [19–21].

We use the “Bayındır Hospital Risk Evaluation Scale for In-hospital Falls of Newborn Infants” in routine monitoring of the babies, along with the monitoring of the vital signs at 3-hour intervals. Besides the precautions taken, nursing support is provided to the babies with high risk scores. A problem encountered in our study was the over-estimation of risky cases, because the high risk threshold was set at a lower level in the scale. We will overcome this handicap by the evaluations performed at each 6 months as indicated in the algorithm, and we will implement new statistical support for our assessments.

Traumas resulting from falling inside the hospital are preventable. Newborn falls may be a serious problem particularly in obstetric clinics of overcrowded hospitals. In an effort to prevent newborn falls before happening, in this prospective study we developed, a scale in accordance with the FMEA. FMEA is a systematic method of resolving and detecting the problems before it starts and aims to properly evaluate a process or product for strengths, weaknesses, potential problem areas, or failure modes, and to prevent problems before they occur. Our results suggest that the most risky situations for newborn falls are the mother with epidural analgesia, holding of the baby at the moment of delivery, and transportation of the baby right after the delivery. After the development of the scale, we implemented all the preventive measures to overcome these risks in our hospital.

Each hospital where delivery operations are carried out, especially those with large patient numbers, must apply necessary precautions suitable for their hospitals to minimize the risks. We believe the scale we developed and the systematic application used in our study will contribute to the literature as to minimizing the risk of trauma of a newborn resulting from falling. 

We think our study can function as a source for obstetric clinics that need to form strategies and clinic manuals to prevent trauma of a newborn resulting from falling. A work strategy for the prevention of in-hospital falls of the newborns should be determined and applied at each hospital.

## Figures and Tables

**Table 1:** Risks determined for in-hospital falls of newborns.

	Risks
1	Patient is admitted to the service to be prepared for the delivery.

2	Birth may occur, by standing up and straining of the patient when the cervix is dilated 5 cm or more.

3	Patient may give birth in the preparation room before the doctor arrives.

4	Birth may occur while the patient is being transferred to the delivery room.

5	Infant may slip down from the hands of the doctor during delivery.

6	Infant may slip down while the umbilical cord is cut.

7	The newborn may fall while it is taken to the newborn care unit for the first medical examination.

8	After having the first medical examination (Apgar scores and general condition fine) the newborn may fall due to turning over of the baby cot while it is being transferred to the baby room to be placed in the incubator in the operating room or in the baby cot in the delivery room.

9	The infant may slip down from the hands of the nurse while it is being transferred to the baby care room in an incubator or baby cot.

10	The infant cared for and dressed is placed in the baby cot and handed to the mother.

11	The mother picks up the infant to breastfeed it. Risk factors regarding the mother are analyzed.

12	The mother who is given patient-controlled analgesia (PCA) drops the baby when she stands up by herself, while no one is near.

13	During the day, the infant is taken to baby room by the baby nurse for checking vital findings, changing diaper and doctor's examinations.

14	Blood sample of the infant is taken to test for phenylketonuria and/or bilirubin on the day of discharge. While final preparations are being made the infant may slip down from the baby care unit.

15	After the mother is given the necessary instructions and while they are being discharged, the infant may slip down from the mother's hands.

**Table 2:** Risk priority numbers determined before and after the preventive measures.

No	Risks	P	S	PF	RPN	Preventive Measures Taken	P	S	PF	RPN
12	The mother who is given patient-controlled analgesia (PCA) drops the baby when she stands up by herself while no one is near.	7	10	5	350	The mother is not allowed to move around by herself. The mother is instructed not to pick up the infant while she is standing. Her doctor is informed. Support of a second person (nurse, accompanying person) is provided when the mother wants to hold the baby to breastfeed/care.	3	10	2	60

5	Infant may slip down from the hands of the doctor during delivery.	4	10	6	240	At vaginal delivery operations, before the delivery a thick towel is placed inside the cuvette of the obstetrical table in order to prevent the infant from falling. After the infant is delivered, the assisting nurse cuts off the umbilical cord while the doctor holds the infant with the right technique.	2	10	2	40

8	After having the first medical examination (apgar scores and general condition fine) the newborn may fall due to turning over of the baby cot while it is being transferred to the baby room to be placed in the incubator in the operating room or in the baby cot in the delivery room.	6	10	4	240	The floor is inspected for dryness. The safety of the equipments that will be used is checked before the transport The baby should be not be hold in the arms, it should be transported by the incubator/cot. When moved around, the cot must be held from the metal portion mounted on the main body (the bassinet portion that is not attached to the main body can slide during movement). When the baby is transported from the cot/incubator to another place, the cot/incubator is held close and at the same level with the bed/equipment. When an elevator is used during the transport of the baby, the height difference between the floors of the elevator and the flat should be checked (height difference may unbalance the cot, the cot may turn over and the baby can fall).	2	10	2	40

2	Birth may occur, by standing up and straining of the patient when the cervix is dilated 5 cm or more.	3	10	6	180	The mother is not allowed to get out of the bed if the cervix is dilated to 5-6 cm. Particularly toilet and all the other needs of the patient are resolved in bed. The expectant is observed by a NST monitor, and when she gets out of her bed the nurse takes notice and checks the patient. The expectant is transferred to obstetrical table at the suitable phase of predelivery (before fetal head comes out of perineum).	1	10	2	20

13	During the day, the infant is taken to baby room by the baby nurse for checking vital findings, changing diaper, and doctor's examinations.	5	9	4	180	The floor of the mother's room is kept dry. Support of a second person is provided. The floor of the baby care room is always kept dry. Bathing of the baby is done in the bathtub of the baby care room. The mother is informed about the falls risk.	2	9	2	36

10	The infant cared for and dressed is placed in the baby cot and handed to the mother.	4	10	4	160	The floor is inspected for dryness. The safety of the equipments that will be used is checked before the transport.	2	10	1	20
						The baby should not be hold in the arms, it should be transported by the incubator/cot.				
						When moved around, the cot must be held from the metal portion mounted on the main body (the bassinet portion that is not attached to the main body can slide during movement).				
						When the baby is transported from the cot/incubator to another place, the cot/incubator is held close and at the same level with the bed/equipment.				

11	The mother picks up the infant to breastfeed it. Risk factors regarding the mother are analyzed.	4	10	4	160	The mother is informed about the falls risk. The mother/accompanying person is reeducated on the risks of falling (Most of the falling incidents occur at night and at dawn). If the pain score of the patient is ≥4, she is not allowed to pick up the infant by herself. The doctor of the mother is instructed. The mother is allowed to pick the infant under supervision and support of a nurse. The doctor of the infant is instructed. The falling risk of the mother is evaluated by the “Adult Fall Scale”. The mother is informed to ask for nursing support whenever she breastfeeds/cares/holds the baby.	2	8	2	36

7	The newborn may fall while it is taken to the newborn care unit for the first medical examination.	3	10	4	120	The baby is placed in the cot prepared before delivery, which has a sterile blanket in it, by the doctor, to prevent the infant from passing from person to person. The doctor of newborn unit and the assisting infant nurse picks up the infant with the cot and take the infant to baby care unit taking the shortest way between the cot and the newborn care unit (open bed).	1	10	1	10

14	Blood sample of the infant is taken to test for phenylketonuria and/or bilirubin on the day of discharge. While final preparations are being made the infant may slip down from the baby care unit.	4	9	3	108	It is not allowed to hand the baby on during care/examinations. A safe working environment is provided. The falling risk that may be caused by the connected equipments is prevented. The floor is inspected for dryness. The safety of the equipments that will be used is checked before the transport. The baby should be not be hold in the arms, it should be transported by the incubator/cot.	1	9	1	9

15	After the mother is given the necessary instructions and while they are being discharged, the infant may slip down from the mother's hands.	3	9	3	81	The safety of the equipments that will be used is checked before the transport. The baby should be not be hold in the arms, it should be transported by the incubator/cot. The mother/ relatives are warned about risks of falling.	2	9	1	18

6	Infant may slip down while the umbilical cord is cut.	2	10	4	80	Sufficient number of qualified personnel attends the delivery (An obstetrician, a neonatologist, a midwife or nurse, a baby nurse).	1	10	1	10
						After the infant is delivered assisting nurse cuts off the umbilical cord while the doctor holds the infant with the right technique.				
						In deliveries performed by cesarean section, the infant is placed on a sterile cloth on the cesarean table and the cord is cut off by clamping. The cord is put in the cot next to the cesarean table by the assisting nurse. The doctor of the newborn unit takes the infant with the cot and places it in the newborn baby care unit. (While taking the infant from the cot to the newborn care unit, the doctor takes the shortest way).				

9	The infant may slip down from the hands of the nurse while it is being transferred to the baby care room in an incubator or baby cot to baby care room.	4	10	2	80	The floor of the mother's room is kept dry. Support of a second person is provided. The floor of the baby care room is always kept dry. The safety of the equipments that will be used is checked before the transport. The baby should be not be hold in the arms, it should be transported by the incubator/cot. When moved around, the cot must be held from the metal portion mounted on the main body (the bassinet portion that is not attached to the main body can slide during movement). When the baby is transported from the cot/incubator to another place, the cot/incubator is held close and at the same level with the bed/equipment.	2	10	1	20

3	Patient may give birth in the preparation room before the doctor arrives.	2	9	4	72	The doctor of the patient is informed as soon as the patient is admitted to the service.	2	9	1	18

4	Birth may occur while the patient is being transferred to the delivery room.	2	7	4	56	As soon as the patient is admitted to the service, preparations are started. The doctor of the patient is informed. The expectant is transferred to obstetrical table at the suitable phase of pre-delivery (before fetal head comes out of perineum). Attendants are called.	1	7	1	7

1	Patient is admitted to the service to be prepared for the delivery.	2	4	2	16	The patient admitted for delivery is immediately directed to the service.	1	4	2	8

P: Probability of detection of an existing effect; S: Severity of effect; PF: Probability of failure; RPN: Risk priority number.
